# Fabrication of Ternary Titanium Dioxide/Polypyrrole/Phosphorene Nanocomposite for Supercapacitor Electrode Applications

**DOI:** 10.3390/molecules29102172

**Published:** 2024-05-07

**Authors:** Seungho Ha, Keun-Young Shin

**Affiliations:** Department of Materials Science and Engineering, Soongsil University 369, Sangdo-ro, Dongjak-gu, Seoul 06978, Republic of Korea; hsho312@naver.com

**Keywords:** phosphorene, polypyrrole, titanium dioxide, chemical oxidative polymerization, supercapacitor

## Abstract

In this paper, we report a titanium dioxide/polypyrrole/phosphorene (TiO_2_/PPy/phosphorene) nanocomposite as an active material for supercapacitor electrodes. Black phosphorus (BP) was fabricated by ball milling to induce a phase transition from red phosphorus, and urea-functionalized phosphorene (urea-FP) was obtained by urea-assisted ball milling of BP, followed by sonication. TiO_2_/PPy/phosphorene nanocomposites can be prepared via chemical oxidative polymerization, which has the advantage of mass production for a one-pot synthesis. The specific capacitance of the ternary nanocomposite was 502.6 F g^−1^, which was higher than those of the phosphorene/PPy (286.25 F g^−1^) and TiO_2_/PPy (150 F g^−1^) nanocomposites. The PPy fully wrapped around the urea-FP substrate provides an electron transport pathway, resulting in the enhanced electrical conductivity of phosphorene. Furthermore, the assistance of anatase TiO_2_ nanoparticles enhanced the structural stability and also improved the specific capacitance of the phosphorene. To the best of our knowledge, this is the first report on the potential of phosphorene hybridized with conducting polymers and metal oxides for practical supercapacitor applications.

## 1. Introduction

Supercapacitors are rapidly advancing in the field of energy storage technology owing to their fast charge–discharge characteristics, high power density, and long lifespan. The electrode materials of supercapacitors are important factors that directly affect the energy and power densities of energy-storage devices [1,2]. Two-dimensional (2D) materials are suitable as electrode materials in supercapacitors because of their thinness, large specific surface area, and mechanical stability, and they can be easily modified and functionalized to enhance their properties [3,4].

In particular, phosphorene is a prominent two-dimensional material with a direct band gap in the range of 0.3–2.0 eV and a carrier transport path of 1000 cm^2^ V^−1^ s^−1^. It has a 2D puckered honeycomb structure obtained by exfoliating multiple layers of black phosphorus composed of phosphorus (P) atoms [5,6]. Because of this unique structural characteristic, the properties of phosphorene can be easily controlled through electric field effects [7,8]. However, it is sensitive to air and moisture reactions; therefore, it has limitations, such as unreliable chemical stability and low cyclic stability, making it less suitable for use as a supercapacitor electrode material [9]. These drawbacks can be circumvented and the poor electrochemical performance can be ameliorated by fabricating phosphorene nanosheets with metal oxides and conducting polymers, as reported in several studies [10,11,12].

Polypyrrole (PPy) is a representative conductive polymer that exhibits pseudocapacitive behavior and is widely used in supercapacitor applications owing to its high specific surface area, specific capacitance, energy density, and low cost [13,14,15]. PPy exhibits conductivity through its conjugated double-bond configuration that allows electrons to move freely within the physical limits of the polymer chain. This enables the efficient storage and release of charges through the doping/de-doping process, thereby enhancing the efficiency of charge storage and discharging [16]. The pyrrole monomer has excellent water solubility and is easily oxidized, enabling easy synthesis through chemical oxidative polymerization. These characteristics make it well-suited for use as a supercapacitor electrode in composite forms [17,18].

Anatase titanium dioxide (TiO_2_) is a prominent metal oxide and promising material for energy storage because of its low cost, low toxicity, natural abundance, and chemical stability [19,20,21,22]. The large surface area of TiO_2_ provides numerous active sites, facilitating substantial charge storage at the electrode-electrolyte interface. Furthermore, it enhances the electrode response rate, resulting in an increased capacity for ion charging. Consequently, TiO_2_ exhibits a stable charging/discharging cycle suitable for long-term use. Thus, the cyclic stability of the ternary composite can be improved using anatase TiO_2_ nanoparticles [23,24,25].

Herein, we describe a novel high-yield method for the fabrication of ternary TiO_2_/PPy/phosphorene nanocomposites with excellent electrochemical performance via ball milling and chemical oxidative polymerization. Amide bond formation between urea-functionalized phosphorene and PPy enhanced the structural stability and electrical conductivity of the ternary nanocomposite. Moreover, the anatase TiO_2_ nanoparticles improved the electrochemical properties and cyclic performance of the nanocomposite when used as an electrode in supercapacitors. The supercapacitive behaviors of the ternary nanocomposites were investigated using different molar ratios of the nanocomposite materials. To the best of our knowledge, this is the first report on the preparation of phosphorene hybridized with PPy and TiO_2_ as a supercapacitor electrode material. The ternary nanocomposite is suitable for use as a practical and cost-effective electrode in energy storage devices.

## 2. Results and Discussion

**Figure 1:**
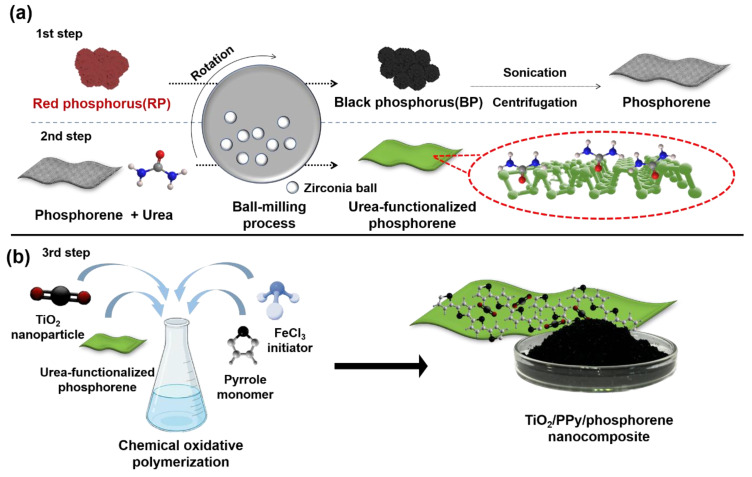
Schematic of the fabrication of ternary TiO_2_/PPy/phosphorene nanocomposite. (**a**) Synthesis of phosphorene nanosheet using ball milling of RP, followed by sonication, and urea-FP via mechanical milling of phosphorene with urea. (**b**) Preparation of the ternary nanocomposite via one-pot chemical oxidative polymerization of pyrrole monomer onto urea-FP with anatase TiO_2_ nanoparticles. The inset shows a petri dish containing large-scale quantities of ternary nanocomposite powder.

Figure 1 shows a schematic of the synthesis of the ternary TiO_2_/PPy/phosphorene nanocomposite. The first step is the conversion of RP into phosphorene, as shown in Figure 1a. Ball milling induces a phase transition from RP to BP by generating high temperatures and pressures inside a jar [11,26,27]. Subsequently, phosphorene is synthesized by the mechanical exfoliation of BP via sonication. The second step involves the preparation of the urea-FP via ball milling with urea and phosphorene. The shear forces generated by the zirconia balls cause urea to be adsorbed onto the phosphorene surface. In particular, the NH_2_ groups of urea can prevent the restacking of the phosphorene nanosheets and aid in the formation of ultrathin nanosheets [28]. In the third step, the TiO_2_/PPy/phosphorene nanocomposite is synthesized via chemical oxidation polymerization (Figure 1b). Pyrrole monomers are polymerized on a 2D phosphorene sheet using FeCl_3_ as the initiator. In this process, TiO_2_ nanoparticles are incorporated into the pyrrole monomer, resulting in the formation of a TiO_2_/PPy nanocomposite structure. Chemical oxidation polymerization has the advantage of the mass production of ternary nanocomposites for one-pot synthesis [29,30]. A black ternary nanocomposite powder was obtained with a yield of 5 g in a single process.

**Figure 2:**
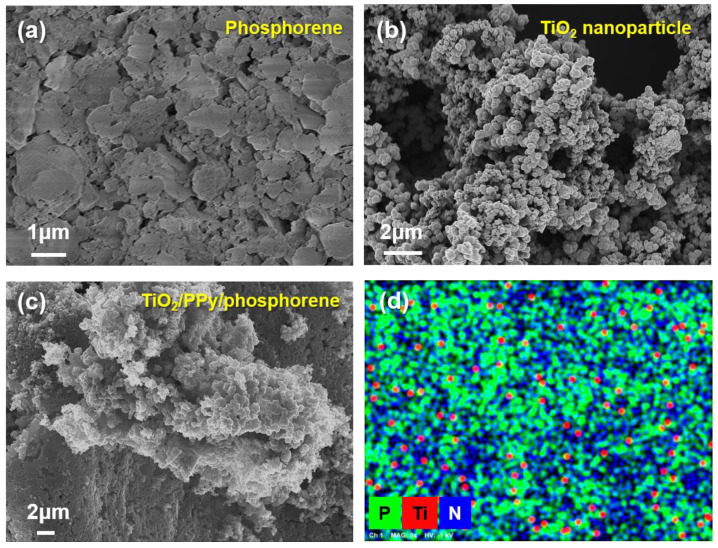
Representative FE-SEM images of (**a**) 2D structure of phosphorene nanosheet, (**b**) anatase TiO_2_ nanoparticle, and (**c**) TiO_2_/PPy/phosphorene nanocomposite. (**d**) EDS image of the ternary nanocomposite.

Figure 2a illustrates the typical 2D structure of phosphorene, where phosphorene nanosheets with widths in the range of 200–800 nm are formed. The surface topographies of BP and phosphorene were analyzed by atomic force microscopy (AFM) to confirm the nanosheet structure (Figure S1). Figure S1a shows multiple stacked and overlapping BP sheets with thicknesses in the range 150–300 nm. The diameters of the phosphorene sheets were 400–500 nm. The thicknesses of the phosphorene sheets were reduced to approximately 1–3 nm, confirming the successful synthesis of few-layer phosphorene (Figure S1b). Notably, the two-step exfoliation process of sonication and urea-assisted ball milling produced ultrathin nanosheets [28]. The diameters of the TiO_2_ nanoparticles were 20–35 nm (Figure 2b). Figure 2c illustrates a nanocomposite structure in which the phosphorene sheets are successfully coated with TiO_2_ and PPy nanomaterials. The EDS image of the ternary nanocomposite shows that P, Ti, and N correspond to phosphorene, TiO_2_, and PPy, respectively (Figure 2d). To confirm the uniformity of the ternary nanocomposite, high-angle annular dark-field scanning transmission electron microscopy (HAADF-STEM) and EDS mapping analysis were conducted for the ternary nanocomposite (Figure S2). The results show that P, N, and Ti are evenly distributed in the UTP nanocomposite, indicating that the nanocomposite structure with phosphorene, PPy, and TiO_2_ was formed uniformly.

**Figure 3:**
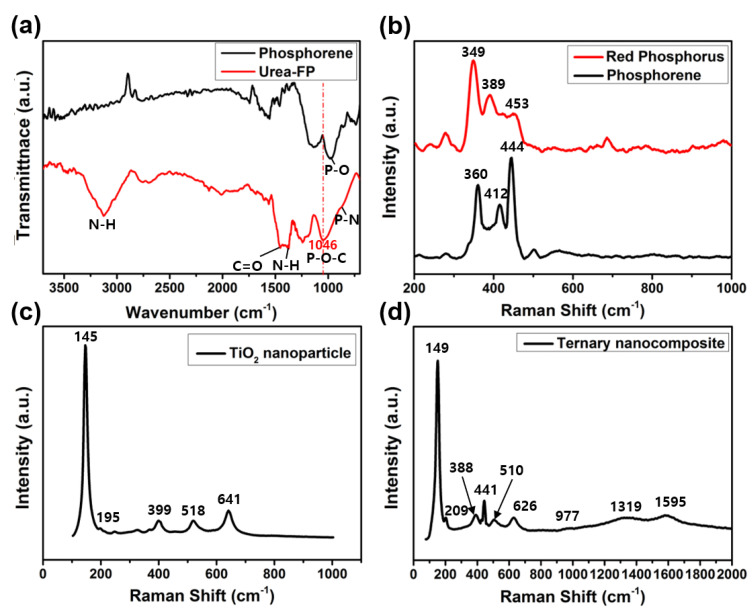
(**a**) FT–IR spectra of urea–FP and phosphorene. Raman spectra of (**b**) RP and phosphorene, (**c**) anatase TiO_2_ nanoparticle, and (**d**) ternary nanocomposite.

Figure 3a shows the FT-IR spectra of phosphorene and urea-FP. Urea-FP exhibits strong, broad peaks at 3121 and 1372 cm^−1^, corresponding to the N–H bond. In addition, the peak at 1046 cm^−1^ is attributed to P–O–C bonds, indicating that phosphorene formed covalent bonds with urea. Figure 3b shows the Raman spectra of the RP and phosphorene. The prominent characteristic peaks of phosphorene are A_1g_, B_2g_, and A_2g_ (360, 412, and 444 cm^−1^) bands; these peaks are shifted slightly relative to those of RP. In RP, the A_1g_ peak (349 cm^−1^) is stronger than that of A_2g_ (461 cm^−1^), whereas in phosphorene, the A_2g_ peak (444 cm^−1^) has a higher intensity than the A_1g_ peak (360 cm^−1^) [31]. The A_1g_/A_2g_ intensity ratio of RP was 2.0, whereas that of phosphorene was 0.7. Furthermore, the B_2g_/A_2g_ intensity ratio of the RP was 0.6, whereas that of phosphorene was 1.5. Unlike RP, the lattice structure of BP exhibits relatively high-frequency vibrational modes owing to its strong covalent bonds [32,33,34]. Consequently, the structures of the RP and BP led to distinguishable Raman peaks. Therefore, the phase transition of RP was confirmed by the Raman peak of phosphorene [35].

The Raman spectra of the anatase TiO_2_ crystal structure with prominent peaks at E_g_ (145, 195, and 641 cm^−1^), B_1g_ (399 cm^−1^), and A_1g_ + B_1g_ (518 cm^−1^) is shown in Figure 3c. The anatase crystal structure had a smaller particle size and larger surface area than the other polymorphs [36]. When anatase TiO_2_ was used as an electrode, these characteristics increased the electron transport and enhanced the electrochemical reaction rate. Therefore, the anatase crystal structure of TiO_2_ is commonly used in energy devices such as supercapacitors. As the size of the TiO_2_ particles is reduced, both the total stored charge and capacitive contribution to the stored charge increase [23].

Figure 3d shows the Raman spectra of the as-synthesized ternary nanocomposites. Peaks are observed at 149, 209, 388, 510, and 626 cm^−1^, which correspond to anatase TiO_2_ nanoparticles. The peak at 441 cm^−1^ originates from the A_2g_ mode of phosphorene. The two broad peaks at 1595 and 1319 cm^−1^ are ascribed to the G and D bands of PPy; the G band is attributed to the π-conjugated structure, while the D band represents the vibration of aromatic rings [21,37]. In addition, the broad peak observed at approximately 980 cm^−1^ corresponds to the combination of the C–H in-plane deformation of PPy and the symmetric stretching of P–O–C [11]. These results suggest that phosphorene nanosheets act as 2D inorganic substrates for the polymerization of PPy and the synthesis of TiO_2_ nanoparticles to prepare ternary nanocomposites.

**Figure 4:**
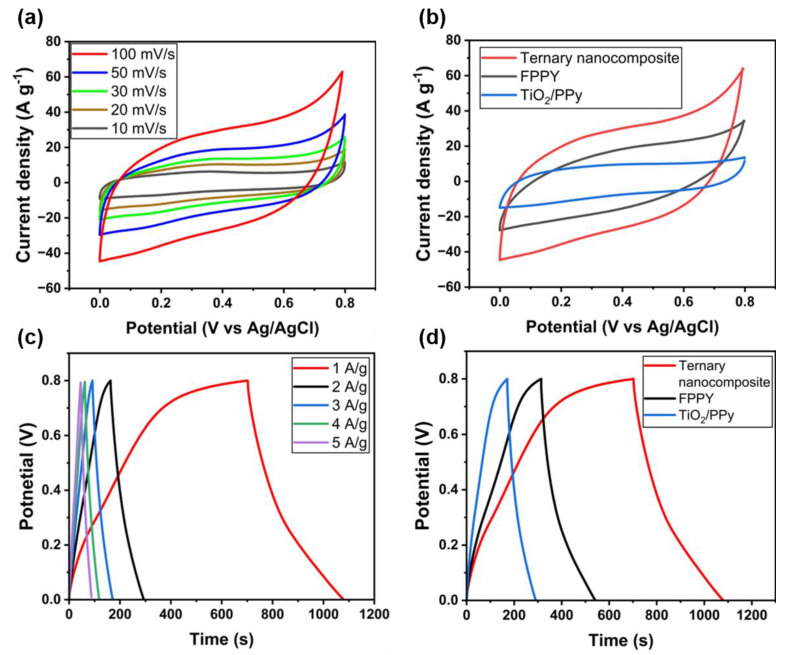
CV curves of (**a**) ternary nanocomposite with different scan rate and (**b**) ternary nanocomposite, FPPY, and TiO_2_/PPy at scan rate of 100 mV s^−1^. GCD curves for (**c**) ternary nanocomposite as a function of current density and (**d**) ternary nanocomposite, FPPY, and TiO_2_/PPy at current density of 1 A g^−1^.

CV measurements were performed to verify the electrochemical behavior of the ternary nanocomposite in the voltage range of 0.0–0.8 V, as shown in Figure 4a. The CV curve shape was stable during the entire change in scan rate from 10 to 100 mV s^−1^. This indicates that the ternary nanocomposite can undergo fast charging, enabling the storage of a considerable amount of charge within a short period [38]. The CV curve shape indicates that the ternary nanocomposite exhibits pseudocapacitive behavior. Generally, phosphorene exhibits pseudocapacitive behavior because of its abundant ion-accessible sites in multiphase active materials [12,39]. Furthermore, anatase TiO_2_ exhibits a pseudocapacitive storage mechanism that enables it to store charge through chemical redox reactions, and the charge stored on its surface can help increase its energy density [21,23]. PPy also exhibits pseudocapacitive properties by adsorbing and storing oxygen and ions via electrochemical reactions [13,40]. Therefore, redox peaks were observed in the electrochemical behavior of ternary nanocomposites [41].

Table S1 lists the ternary nanocomposites with various specific capacitances according to the ratios of the components. Specific capacitance ($C_{sp}$) was calculated using the following equation:

$$C_{sp}=\frac{A}{2mv∆V},$$

where $C_{sp}$ is the specific capacitance, ∆V is the potential range (V), v is the scan rate (V/s), *A* is the area of the CV curve (${cm}^{2}$), and m is the mass of the active material. TiO_2_/PPy/phosphorene with a ratio of 1:1:1 exhibits a specific capacitance of 388 F g^−1^. The highest capacitance value of 502.6 F g^−1^ was achieved with a composite ratio of 60% pyrrole, which was identified as the optimized ternary nanocomposite for supercapacitor electrode applications.

Figure 4b presents a comparison of the specific capacitances of the various types of nanocomposites. In the CV data, the integrated area represents the total amount of charge involved in the electrochemical reaction during a scan and can be used to calculate the specific capacitance and other electrochemical properties. The integral area of the ternary nanocomposite was larger than those of FPPY and TiO_2_/PPy. This indicates that the capacitive behavior of PPy was significantly improved by the addition of phosphorene nanosheets and TiO_2_ nanoparticles.

Figure 4c illustrates the GCD curves of the ternary nanocomposite at various current densities in the range of 1–5 A g^−1^. The specific capacitances for the ternary nanocomposite, FPPY, and TiO_2_/PPy were obtained from GCD at a current density of 1 A g^−1^ (Figure 4d). Discharge-specific capacitance was calculated using the following equation:

$$C_{sp}=\frac{I\times ∆t}{∆V\times m},$$

where I is the discharge current and ∆t is the discharge time in h. The specific capacitances of TiO_2_/PPy and FPPY were 150 and 286.25 F g^−1^, respectively. Considering that the specific capacitance of the ternary nanocomposite was 481.25 F g^−1^, the results obtained from the GCD and CV curves correlate well. The significant improvement in the electrochemical behavior of the ternary nanocomposite can be ascribed to the enhanced electrical conductivity due to the addition of the metal oxide and conducting polymer to the phosphorene nanosheets.

Table 1 indicates that the energy density (*E*) of the ternary nanocomposite is 153.92 Wh kg^−1^ at a current density of 1 A g^−1^, which is much higher than that of TiO_2_/PPy and FPPY. The *E* value was calculated using the following equation:

$$E=\frac{C_{sp}\times {\left(∆V\right)}^{2}}{2}$$

Table 2 indicates that the phosphorene-based ternary nanocomposite exhibits a better specific capacitance than other similar nanocomposites investigated in previous studies. In particular, the capacitance of the ternary nanocomposite was approximately 22% higher than that of the graphene-based composites. Moreover, it demonstrated that the addition of TiO_2_ nanoparticles resulted in a 22% increase in the capacitance.

**Figure 5:**
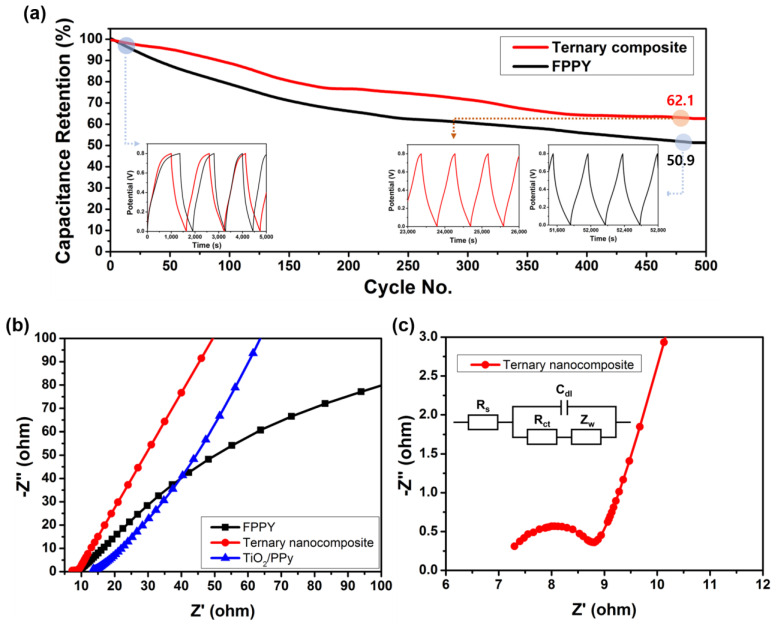
(**a**) Capacitance retention of ternary nanocomposite and FPPY. The insets show the charge/discharge profile at the initial and 500th cycle. The capacitance retentions were characterized at a current density of 1 A g^−1^. (**b**) Nyquist plot of ternary nanocomposite, FPPY, and TiO_2_/PPy-based supercapacitors. (**c**) Magnified Nyquist plot of ternary nanocomposite.

The cycling performance of the ternary nanocomposite was evaluated, and it exhibited a capacitance retention of 62.1% after 500 cycles with a current density of 1 A g^−1^ (Figure 5a). After 500 cycles at the same current density, FPPY demonstrated a capacitance retention of 50.9%. It can be observed that stable cycling is achieved, reaching 0.8 V even after 500 cycles. Furthermore, the ternary nanocomposite exhibited a capacitance retention of 63.7% after 500 cycles with a current density of 10 A g^−1^ (Figure S3). Notably, after 1500 cycles at the same current density, it demonstrated a capacitance retention of 53%. According to a study by Kim et al., a synthesized composite consisting of phosphorene and PPy exhibited a cyclic performance of 56.5% after 500 cycles [11]. The synthesized ternary nanocomposite in this study shows three times higher cyclic stability at the same retention value.

Figure 5b,c display the EIS results of ternary nanocomposite, FPPY, and TiO_2_/PPy, in the frequency range of 0.1 to 100 kHz. The contact resistance (*R_s_*) of the ternary nanocomposite was 7.00 Ω. In contrast, the *R_s_* values of FPPY and TiO_2_/PPy were 8.80 and 13.87 Ω, respectively. This indicated that the ternary nanocomposite had the highest electrical conductivity. Namely, the addition of TiO_2_ nanoparticles enhanced the charge carrier mobility as well as the cyclic stability of the ternary nanocomposite-based supercapacitor.

## 3. Experimental Section

### 3.1. Materials

Red phosphorus (RP) was purchased from Duksan chemicals. Titanium isopropoxide (TTIP, 97%), urea (≥98%), nitric acid (70%), polyvinylidene fluoride (PVDF), iron (III) chloride (FeCl_3_), and carbon black were obtained from Sigma-Aldrich. Absolute ethanol and *N*–methyl–2–pyrrolidone (NMP) (99.5%) were purchased from Samchun Chemicals.

### 3.2. Synthesis of Phosphorene from RP

Ball-milling techniques were used for the phase transition from RP to black phosphorus (BP). RP (1 g) and zirconia balls (0.5 mm diameter, 20 g) were placed in a zirconia bowl. Ball milling was conducted for 48 h at 550 rpm and paused for 5 min every 25 min to prevent overheating. The suspension was separated from the ball using a sieve. After centrifugation at 9000 rpm, the obtained precipitate was dried overnight at room temperature (RT) in a vacuum oven. The as-prepared BP was dissolved in an ethanol solution (40 vol% in water). The solution was sonicated for 2 h using ultrasonication. After centrifugation at 8000 rpm to remove the solvent and drying overnight, the phosphorene powder was collected.

### 3.3. Synthesis of Urea-Functionalized Phosphorene (Urea-FP)

The prepared phosphorene (0.3 g) and urea (0.6 g) were poured into a bowl and ball-milled for 2 h at 550 rpm. The suspension was separated from the ball using a sieve, and the solvent was removed by centrifugation. The obtained sediment was dried overnight, and the urea-FP powder was collected.

### 3.4. Preparation of Anatase TiO_2_ Nanoparticles

Anatase TiO_2_ nanoparticles were synthesized from the TTIP precursors using the sol-gel method. First, nitric acid (0.13 mL) was dissolved in 100 mL distilled (DI) water. TTIP (1 mL) dissolved in 9 mL of absolute ethanol (10 vol/vol%) was added dropwise to the first solution. Subsequently, the solution was stirred at 650 rpm and heated to 70 °C for 24 h. After the reaction, the solvent was removed by vacuum filtration. The obtained powder was ground into a fine powder in a mortar and heat-treated in a furnace at 500 °C.

### 3.5. Fabrication of Ternary TiO_2_/PPy/Phosphorene Nanocomposite

A ternary TiO_2_/PPy/phosphorene nanocomposite was prepared via chemical oxidative polymerization. The pyrrole monomer (0.13 M) was dispersed in DI water (60 mL). The as-prepared urea-functionalized phosphorene (0.1 g) and TiO_2_ nanoparticles (0.1 g) were then added to the solution. Subsequently, FeCl_3_ (2.11 g) was dissolved in DI water (40 mL) and added dropwise. The solution was stirred for 8 h at 0–5 °C. The obtained nanocomposites were washed with DI water to remove impurities or FeCl_3_ and dried overnight at RT in a vacuum oven.

### 3.6. Fabrication of TiO_2_/PPy Nanocomposite

The pyrrole monomer (0.13 M) was dispersed in DI water (60 mL). TiO_2_ nanoparticles (0.1 g) were then added to the solution. An aqueous solution of FeCl_3_ was then added dropwise and allowed to react for 1 h. The solution was stirred for 8 h at 0–5 °C. The obtained TiO_2_/PPy nanocomposites were washed with DI water to remove impurities or FeCl_3_ and dried overnight at RT in a vacuum oven.

### 3.7. Fabrication of Urea-FP/PPy (FPPY) Nanocomposite

Urea-FP was dispersed in DI water (100 mL, 1.0 mg mL^−1^) and sonicated for 1 h. Subsequently, the pyrrole monomer (0.5 mL) was dissolved in the solution. The resultant solution was stirred at 650 rpm and RT for 2 h. An aqueous FeCl_3_ solution was then added dropwise and allowed to react for 1 h. The obtained FPPY nanocomposites were washed with DI water to remove impurities or FeCl_3_ and dried overnight at RT in a vacuum oven.

### 3.8. Electrochemical Measurement of Nanocomposites for Supercapacitor Electrode

Electrochemical measurements were performed using a three-electrode cell with a 1M H_2_SO_4_ aqueous solution as the electrolyte, a Pt wire as the counter electrode, and Ag/AgCl as the reference electrode. The ternary nanocomposites, TiO_2_/PPy and FPPY, were drop-cast onto a glassy carbon working electrode by mixing the active material (80 wt.%), PVDF (10 wt.%), carbon black (10 wt.%), and NMP. Electrochemical measurements were conducted on the ternary nanocomposite to evaluate its supercapacitor performance.

### 3.9. Characterization

Morphological images were obtained using field emission-scanning electron microscopy (FE-SEM; GEMINISEM 300, Carl Zeiss, Oberkochen, Germany), energy dispersive X-ray spectrometry (EDS; XFlash 6-30, Bruker, Ettlingen, Germany), transmission electron microscopy (TEM; F200X G2, Talos, MA, USA), and atomic force microscopy (AFM; NX10, Park Systems, Suwon, Republic of Korea). Raman spectroscopy and Fourier-transform infrared spectroscopy (FT-IR) were conducted using an inVia Raman microscope (Renishaw, Wotton-under-Edge, UK) and a VERTEX70 (Bruker, Ettlingen, Germany), respectively. A ZIVE SP2 electrochemical workstation (WonATech, Seoul, Republic of Korea) was used for the electrochemical characterization, including cyclic voltammetry (CV), electrochemical impedance spectroscopy (EIS), and galvanostatic charge and discharge (GCD) tests.

## 4. Conclusions

An effective method for the fabrication of TiO_2_/PPy/phosphorene nanocomposites with high electrochemical performance was demonstrated via ball milling and chemical oxidative polymerization. Phosphorene nanosheets acted as 2D inorganic substrates for the ternary nanocomposite, and the PPy and TiO_2_ nanoparticles exhibited enhanced charge-carrier mobility and cyclic stability. These properties resulted in the ameliorated electrochemical performance and pseudocapacitive behavior of the hybrid nanomaterials. The ternary nanocomposite shows promise as a key component in supercapacitors that can be rapidly charged and discharged over a long period, paving the way for new possibilities for energy storage applications.

## Figures and Tables

**Table 1:** Specific capacitance and energy density of ternary nanocomposite, FPPY, and TiO_2_/PPy-based supercapacitor electrode.

Samples	Specific Capacitance (F g^−1^)	Energy Density (Wh kg^−1^)
Ternary nanocomposite	502.6	153.92
FPPY	286.25	91.6
TiO_2_/PPy	150.0	48.0

**Table 2:** Comparison of specific capacitance values for various nanocomposite-based supercapacitor electrodes.

Samples	CV (F g^−1^)	GCD (F g^−1^)	Ref.
Ternary nanocomposite	502.6 (at 10 mV s^−1^)	481.3 (at 1 A g^−1^)	Present work
TiO_2_@PPy/rGO	394.2 (at 10 mV s^−1^)	410.1 (at 1 A g^−1^)	[42]
PPy-PT/TiO_2_	–	271.8 (at 1 A g^−1^)	[43]
Graphene/TiO_2_/PPy	431.2 (at 10 mV s^−1^)	–	[30]
R–BP/SPC	–	364.5 (at 0.5 A g^−1^)	[44]
FPPY	411.5 (at 2 mV s^−1^)	–	[11]

## Data Availability

The original contributions presented in the study are included in the article/Supplementary Materials, further inquiries can be directed to the corresponding author/s.

## Supplementary Materials

The following supporting information can be downloaded at: https://www.mdpi.com/article/10.3390/molecules29102172/s1, Figure S1: Representative AFM images and height profile of (**a**) black phosphorus and (**b**) phosphorene nanosheet; Figure S2: (**a**) HAADF-STEM and (**c**,**d**) EDS mapping images of ternary nanocomposite: (**b**) P (mint), (**c**) N (green), (**d**) Ti (purple); Table S1: Specific capacitances of ternary nanocomposite-based supercapacitors with different molar ratio of the components; Figure S3: Cycle stability of ternary nanocomposite-based supercapacitor. The capacitance retentions were characterized at a current density of 10 A g^−1^.
