# Supplementary material for: Fabrication of Ternary Titanium Dioxide/Polypyrrole/Phosphorene Nanocomposite for Supercapacitor Electrode Applications
Source: Molecules. 2024 May 7;29(10):2172. doi: 10.3390/molecules29102172 (PMC11124188; doi:10.3390/molecules29102172)
Supplement: Supplementary file 1 [file molecules-29-02172-s001.zip › molecules-2984665-supplementary.pdf]

Supplementary Materials

Fabrication of Ternary Titanium Dioxide/Polypyrrole/ Phosphorene Nanocomposite for Supercapacitor Electrode Applications

Seungho Ha and Keun-Young Shin *

Department of Materials Science and Engineering, Soongsil University 369, Sangdo-ro, Dongjak-gu, Seoul 06978, Republic of Korea; hsho312@naver.com

* Correspondence: skykek@ssu.ac.kr

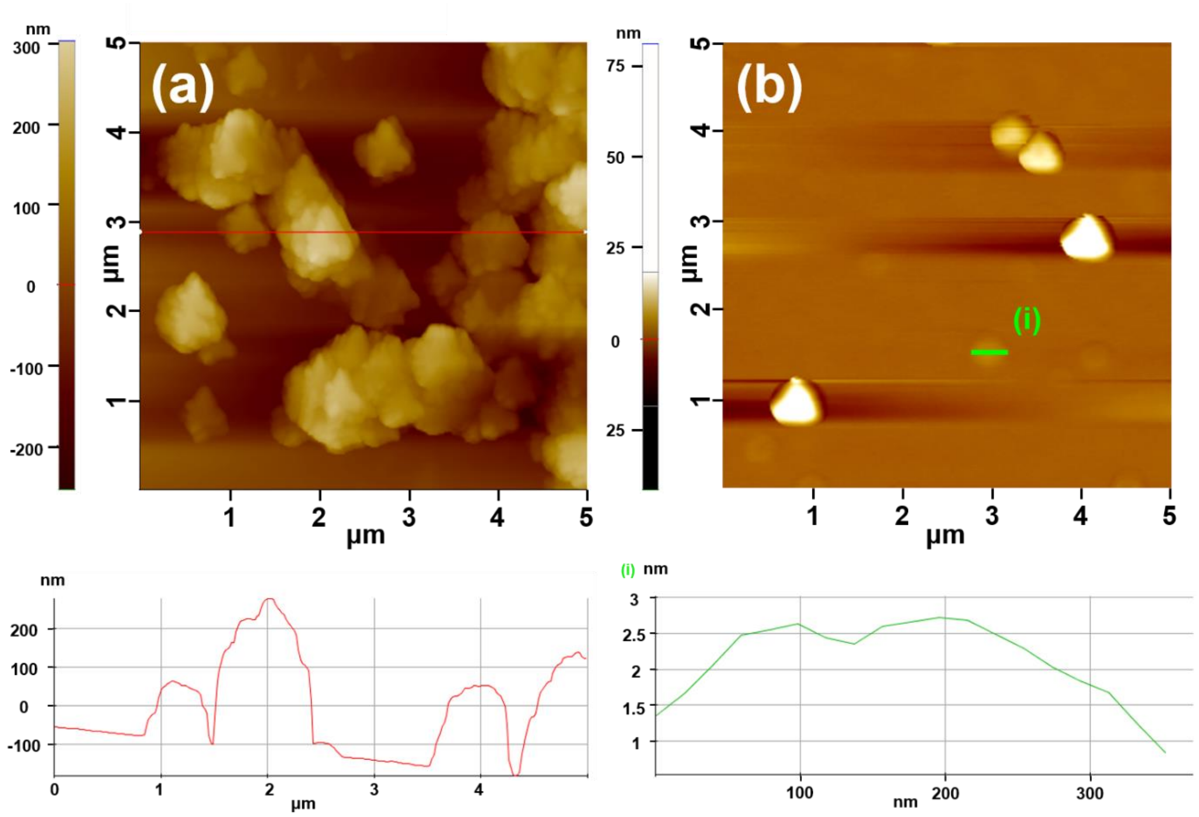

Figure S1. Representative AFM images and height profile of (a) black phosphorus and (b) phosphorene nanosheet.

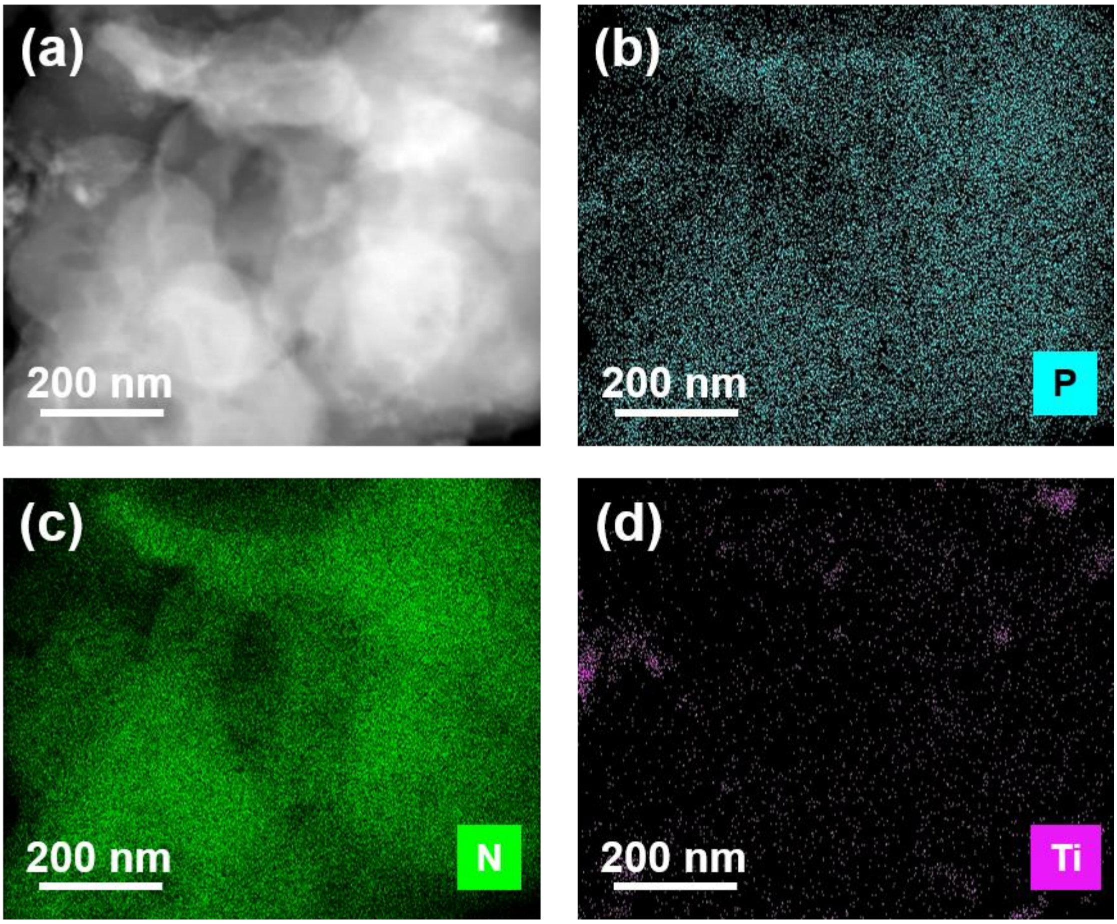

Figure S2. (a) HAADF-STEM and (c-d) EDS mapping images of ternary nanocomposite: (b) P (mint), (c) N (green), (d) Ti (purple).

Table S1. Specific capacitances of ternary nanocomposite-based supercapacitors with different molar ratio of the components.

	Mol(%)			Specific capacitance (Fg^{-1})
	Urea-FP	Pyrrole monomer	TiO_2 nanoparticle	
**Sample 1**	28%	59%	13%	**502.6**
**Sample 2**	56%	33%	11%	**400**
**Sample 3**	34%	33%	33%	**388**
**Sample 4**	14%	83%	3%	**203.12**
**Sample 5**	2%	95%	3%	**132.8**

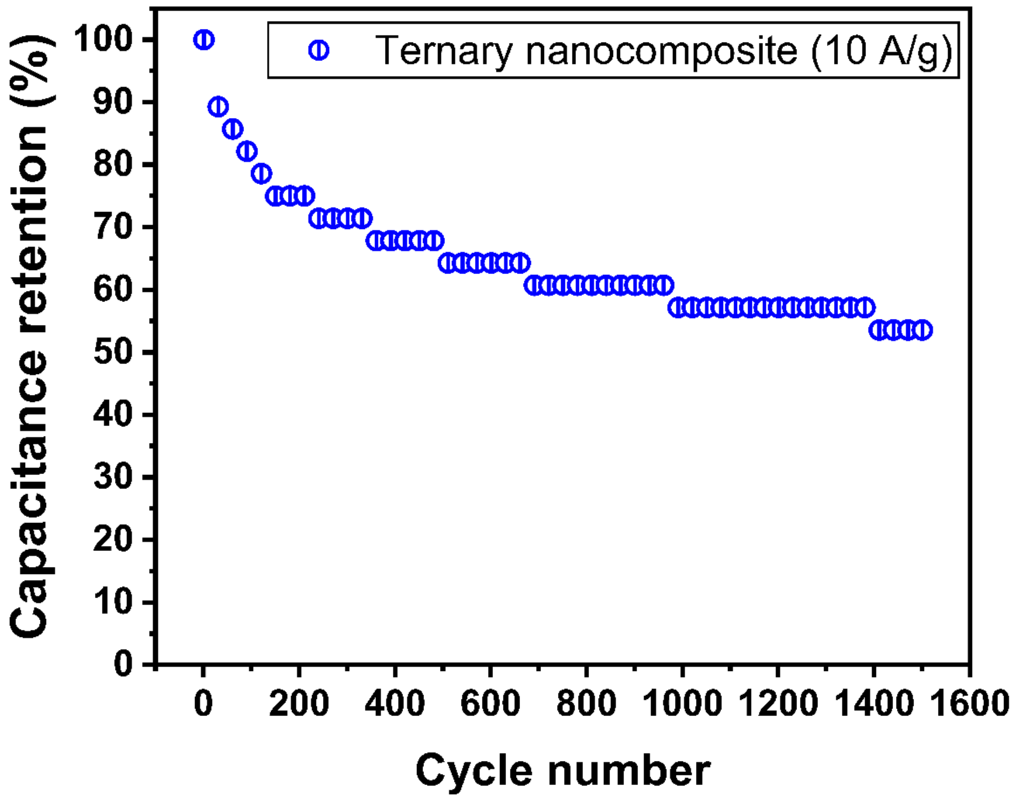

Figure S3. Cycle stability of ternary nanocomposite-based supercapacitor. The capacitance retentions were characterized at a current density of 10 A g^{-1}.
